# Fronto-parietal and fronto-temporal theta phase synchronization for visual and auditory-verbal working memory

**DOI:** 10.3389/fpsyg.2014.00200

**Published:** 2014-03-18

**Authors:** Masahiro Kawasaki, Keiichi Kitajo, Yoko Yamaguchi

**Affiliations:** ^1^Department of Intelligent Interaction Technology, Graduate School of Systems and Information Engineering, University of TsukubaTsukuba, Japan; ^2^Rhythm-based Brain Information Processing Unit, RIKEN BSI-TOYOTA Collaboration CenterIbaraki, Japan; ^3^Laboratory for Advanced Brain Signal Processing, RIKEN Brain Science InstituteSaitama, Japan; ^4^Neuroinformatics Japan Center, RIKEN Brain Science InstituteSaitama, Japan

**Keywords:** working memory, electroencephalogram, synchronization, visual, auditory

## Abstract

In humans, theta phase (4–8 Hz) synchronization observed on electroencephalography (EEG) plays an important role in the manipulation of mental representations during working memory (WM) tasks; fronto-temporal synchronization is involved in auditory-verbal WM tasks and fronto-parietal synchronization is involved in visual WM tasks. However, whether or not theta phase synchronization is able to select the to-be-manipulated modalities is uncertain. To address the issue, we recorded EEG data from subjects who were performing auditory-verbal and visual WM tasks; we compared the theta synchronizations when subjects performed either auditory-verbal or visual manipulations in separate WM tasks, or performed both two manipulations in the same WM task. The auditory-verbal WM task required subjects to calculate numbers presented by an auditory-verbal stimulus, whereas the visual WM task required subjects to move a spatial location in a mental representation in response to a visual stimulus. The dual WM task required subjects to manipulate auditory-verbal, visual, or both auditory-verbal and visual representations while maintaining auditory-verbal and visual representations. Our time-frequency EEG analyses revealed significant fronto-temporal theta phase synchronization during auditory-verbal manipulation in both auditory-verbal and auditory-verbal/visual WM tasks, but not during visual manipulation tasks. Similarly, we observed significant fronto-parietal theta phase synchronization during visual manipulation tasks, but not during auditory-verbal manipulation tasks. Moreover, we observed significant synchronization in both the fronto-temporal and fronto-parietal theta signals during simultaneous auditory-verbal/visual manipulations. These findings suggest that theta synchronization seems to flexibly connect the brain areas that manipulate WM.

## Introduction

Working memory (WM) consists of not only a short-term maintenance system, but also a central executive system that manipulates the maintained representation (Baddeley, 2007). Previous studies have proposed that the executive system is associated with the prefrontal area, whereas the maintenance system is distributed in more posterior sensory areas, such as occipito/parietal areas for visual WM and temporal areas for auditory-verbal WM (Smith and Jonides, 1999; Rowe et al., 2000; Wager and Smith, 2003; Sakai and Passingham, 2004). Recent human electroencephalography (EEG) studies have demonstrated an important role for large-scale phase synchronization in WM (Fries, 2005; Klimesch et al., 2008), as such synchronous neural oscillations are thought to link multiple brain regions dynamically (Engel and Singer, 2001; Varela et al., 2001; Ward, 2003). In fact, the theta-range (4–8 Hz) phase synchronizations between the prefrontal cortex and the relevant cortical areas form the executive functions that link the storage systems during the manipulation of mental representations (Kawasaki et al., 2010). However, there remain open questions regarding how relevant information is flexibly selected for manipulation in the mind and how irrelevant information is stored during the mental manipulations that are required to perform multiple tasks simultaneously.

To investigate brain-network dynamics, we measured EEG signals during a single or dual task for two sensory modalities and analyzed phase synchronization between distant cortical areas. Here, we used two types of WM manipulation tasks: an auditory-verbal WM task, which required the mental calculation of numbers presented through an auditory-verbal stimulus; and a visual WM task, which required the participants to move a spatial location in a mental representation in accordance with a visual stimulus. In addition to the two single WM tasks, each subject performed the two tasks sequentially or simultaneously, as dual tasks.

We conducted region-of-interest analyses, because we had previously identified the representative electrodes of prefrontal, auditory-verbal, and visual areas by analyzing EEG data from the same two single WM tasks (Kawasaki et al., 2010). The theta amplitudes on the frontal and visual electrodes and on the frontal and auditory-verbal electrodes were enhanced during the manipulation of visual and auditory-verbal representations, respectively. Moreover, theta phase synchronization between the electrodes was observed for the relevant WM tasks.

## Materials and methods

### Subjects

Fourteen healthy volunteers (10 males and 4 females; mean age = 27.92 ± 6.76 years, range, 21–41 years; 13 right-handed individuals) participated in this experiment. The subjects reported via subjective questionnaires regarding having normal visual acuity (with or without correction), hearing, and motor abilities. All subjects gave written informed consent prior to participation in this study. The study was approved by the RIKEN Ethics Committee (in accordance with the Declaration of Helsinki).

### Experimental procedure

Each subject completed five separate sessions in a random order among subjects: one auditory-verbal WM condition, one visual WM condition, two sequential dual WM condition, and one simultaneous dual WM condition. Throughout the sessions, the subjects faced a computer screen with headphones.

**Figure 1:**
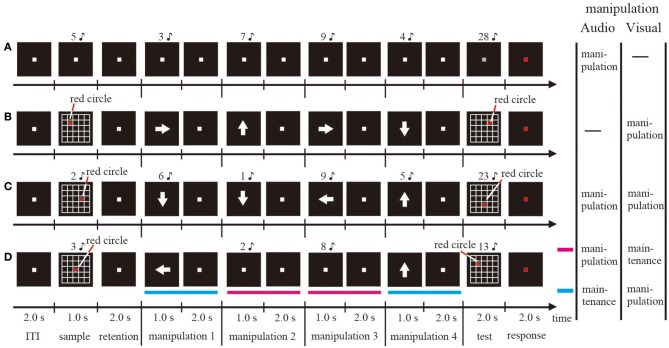
**Schematic illustrations of one trial sequence for the visual single WM (A), the auditory-verbal single WM (B), the simultaneous WM (C), and the sequential WM (D) tasks**.

For the auditory-verbal WM condition (Figure 1A), at the beginning of each trial, a word indicating a 1-digit number was presented as the auditory-verbal stimulus to both ears for 1 s via the headphones (sample stimulus). Subjects were required to memorize and maintain the presented number with rehearsal in their minds. After a 2-s retention interval, another 1-digit number was presented as the audio stimulus for 1 s, and then the subjects were asked to update and maintain the number in their minds by adding the presented number and the maintained number for 2 s. Subjects were required to repeat the mental addition four times, and then determine whether the total number that they calculated mentally matched a probe audio stimulus presented after a white fixation point on a gray PC display (test stimulus). In half of the trials, the test stimulus matched the total number. In the remaining trials, the wrong total number was presented as the probe stimulus by replacing one of the four numbers presented with a different number.

For the visual WM condition (Figure 1B), at the beginning of each trial, 5 × 5 gridded squares and a red circle included within one of those squares were presented for 1 s on the computer screen as the visual stimulus (sample stimulus). Subjects were required to memorize and then maintain the position of the red circle for 2 s after the visual stimulus disappeared. Subsequently, a white arrow designating the direction toward which the subjects had to move the red circle in their minds was presented at the center of the screen for 1 s. The subjects manipulated the mental representations for 2 s. The direction of the arrow was upward, downward, rightward, or leftward. Similar to the audio WM condition, the subjects were required to repeat the mental manipulation four times, and then determine whether the position of the red circle that they moved mentally matched a probe visual stimulus (test stimulus). In half of the trials, the probe stimulus matched the mental representation. In the remaining trials, the wrong probe was presented by changing only the fourth direction of the moving from the actual direction. The size of the red circle and the gridded squares were 1 × 1° and 5 × 5° (1 × 1° per square), respectively.

For the sequential dual WM condition (Figure 1D), the visual and audio stimuli described above were presented simultaneously on the computer screen and via the headphones, respectively (sample stimulus). Subjects were required to memorize both the auditory-verbal and visual stimuli and, after the retention interval, manipulate either the auditory-verbal or the visual representation. If an arrow was presented on the screen, subjects performed the visual WM task; if a number was presented via the headphones, they performed the auditory-verbal WM task. Each task was performed randomly twice. The subjects were not aware of the sequences of auditory-verbal and visual WM tasks. After a total of four manipulations, the auditory-verbal and visual stimuli were presented simultaneously and subjects were required to judge whether they were identical to the manipulated mental representation for both auditory-verbal and visual WM tasks or not (test stimulus). In half of the trials, both the auditory-verbal and visual test stimuli matched the mental representations. In the remaining trials, the wrong probe for either auditory-verbal or visual stimuli was presented, similar to what was observed in the single auditory-verbal and visual WM conditions.

For the simultaneous dual WM condition (Figure 1C), the procedures of sample display, retention interval, and test stimulus were identical to those used in the sequential dual WM condition, with the exception that the auditory-verbal and visual stimuli were presented simultaneously and the participants were asked to perform both the auditory-verbal and visual WM tasks during each manipulation period.

In all conditions, subjects were asked to indicate, by pressing a button, whether the stimulus was correct or not while the fixation point was kept red for 2 s. Each session consisted of 24 trials. The duration of the inter-trial interval (ITI) was 2 s (Figure 1). The ITI was defined as the baseline period in this study. In a given session, one condition was being tested. All participants underwent a training session before the corresponding EEG measurement session. The stimulus was generated on a Windows computer using Matlab 7.5.0 (Mathworks, Inc., Natick, MA) with the Psychophysics Toolbox extension. The sound of each number was highly distinctive.

### EEG recordings and analyses

EEG was recorded continuously from 62 scalp electrodes (Ag/AgCl) embedded in an electrode cap (Easy Cap; EASYCAP GmbH, Germany) and in accordance with the placement of the international 10/10 system. EEG signals were referenced digitally to the averaged recordings from the right and left earlobes. Electrode impedance was maintained below 14.6 kΩ. Electrooculography (EOG) was recorded from electrodes that were placed above and below the left eye, to monitor eye blinks or vertical eye movements. EOG electrodes placed 1 cm lateral from the right and left eyes monitored horizontal eye movements. The EEG and EOG signals were amplified using the Neuroscan system (Neuroscan, USA). The sampling rate was 500 Hz.

EEG data were preprocessed by first segmenting the EEG data into 5-s epochs (with 1-s pre-manipulation, 3-s manipulation, and 1-s post-manipulation periods; 2500 time points in total). Epochs containing artifacts caused by blinks or eye movements were detected from the EOG and EEG data using an amplitude criterion (±100 μV) and were excluded from further analyses.

Next, to identify cortical activity with reduced effects of volume conduction, we applied a current source density transformation to the voltage distribution on the surface of the scalp using the spherical Laplace operator (Perrin et al., 1989; Kayser and Tenke, 2006). Finally, to identify the time-frequency phases, we applied wavelet transforms using Morlet's wavelet function (Tallon-Baudry et al., 1997).

We used Morlet's wavelets for the high time and frequency resolutions, which allowed a better observation of transitions in both low- and high-frequency oscillations (Herrmann et al., 2005). The phase for each time point in each transcranial magnetic stimulation (TMS) application was the arctangent of the results of the convolution of the original EEG signal *s*(*t*) with a complex Morlet's wavelet function *w*(*t*, *f*):

$$w(t,f)=\sqrt{f}\exp\left(-\frac{t^{2}}{2\sigma_{t}^{2}}\right)\;\exp\;\left(i2\pi ft\right)$$

where σ_*t*_ is the standard deviation of the Gaussian window. The wavelet used here was roughly characterized by the number of cycles n_*co*_ within a 6σ_*t*_ interval (Lachaux et al., 2000), which contains about 99.7% of the power of the Gaussian window. We chose *n*_*co*_ = 3 (= 6*f*σ_*t*_), with *f* ranging from 1 to 20 Hz in 1-Hz steps.

### Phase synchronization index (PSI)

To identify the phase relations between any two electrodes, the PSI for each time point and each electrode pair was defined by the following equation:

$$PSI_{jk}(t,\;f)=\sqrt{{\left(\sum_{i=1}^{N}\cos(\Delta \phi_{jk}(i,\;f))/N\right)}^{2}+\;{\left(\sum_{i=1}^{N}\sin\left(\Delta \phi_{jk}(i,\;f)\right)/N\right)}^{2}}$$

where Δ Φ_*j k*_(*t*, *f*) is the phase difference between *j*th and *k*th electrodes and the number of time points *N* with an interval of 1 s is 500.

To evaluate the task-related PSI changes, we applied a bootstrap calculation to the PSIs of the individual subjects and compared the virtual PSI data during the tasks [φ^*A*^_*j k*_ (*t*, *f*)] and baseline data of the ITI period [φ^*B*^_*j k*_ (*t*, *f*)], as follows:

$$\begin{array}{l} \varphi_{jk}^{A*}(t,\;f)=\varphi_{jk}^{A}(t,\;f)-{\bar{\varphi }}_{jk}^{A}(f)+{\bar{\varphi }}_{jk}(f)\\ \varphi_{jk}^{B*}(t,\;f)=\varphi_{jk}^{B}(t,\;f)-{\bar{\varphi }}_{jk}^{B}(f)+{\bar{\varphi }}_{jk}(f) \end{array}$$

where φ^A*^_j k_ (*t*, *f*) and φ^B*^_j k_ (*t*, *f*) represent the original PSI, φ ^*A*^_*j k*_ (*f*), φ ^*B*^_*j k*_ (*f*), and φ_*j k*_(*f*) represent the means of φ^*A*^_*j k*_ (*t*, *f*), φ^*B*^_*j k*_ (*t*, *f*), and all of the data, respectively. We performed a 2-sample *t*-test using the 2000 bootstrapped re-samples of each time point for individual subjects (Kawasaki et al., 2010).

We calculated the *Z*-values which mean “the degrees about whether differences are significant or not” by using the non-parametric Wilcoxon signed-rank test for the different normalized PSI values. The null hypothesis is that the difference of the representative PSI values between the events equals to zero. If the *Z*-value is near zero, the different PSI values between the events are not significant (the null hypothesis is not rejected). On the other hand, if the *Z*-value is larger than the statistical threshold, the difference in PSI values between the events is statistically significant (the null hypothesis is rejected). To test if the significance results from a chance, we repeated the Wilcoxon signed-rank tests for 2000 times with the bootstrapped resample data of each time point for individual subjects.

Finally, we tested whether the mean of the distribution of the 2000 resampled *Z*-values is zero or not by using the sign test against the null hypothesis that the mean of *Z*-values equals to zero. If the *Z*-value is near zero, the difference in PSI values between the events is not significant (the null hypothesis is not rejected). On the other hand, if the *Z*-value is larger than the significance threshold, it is no coincidence that the difference in PSI values between the events is statistically significant (the null hypothesis is rejected).

## Results

### Behavioral results

The subject-averaged accuracy rates were 95.2 ± 1.6, 97.3 ± 1.2, 89.9 ± 1.9, and 94.9 ± 1.1% (mean accuracy rate ± s.e.m.) for auditory-verbal (A), visual (V), simultaneous dual (Sim), and sequential dual (Seq) WM conditions, respectively (Table1).

**Table 1:** **Mean accuracy rate ± standard error mean (s.e.m.) for each condition**.

	**Audio**	**Visual**	**Sim. dual**	**Seq. dual**
Mean accuracy rate ± s.e.m. (%)	95.2 ± 1.6	97.3 ± 1.2	89.9 ± 1.9	94.9 ± 1.1

A one-way repeated measures ANOVA revealed a significant main effect of the task conditions [*F*_(3, 39)_ = 5.18, *P* < 0.005]. There was a significant difference between visual WM and sequential dual WM conditions but not among other combinations. The *post-hoc* analyses (Tukey HSD test) revealed HSD-values as follows: A vs. V, HSD = 0.021; A vs. Sim, HSD = −0.042; A vs. Seq, HSD = −0.003; V vs. Sim, HSD = −0.063, *P* < 0.01; V vs. Seq, HSD = −0024; Sim vs. Seq, HSD = 0.039.

### PSI results

This study selected the AF3, P5, and Pz electrodes as representative frontal, temporal, and parietal electrodes, respectively, because the AF3-P5 and AF3-Pz pairs showed clear synchronizations during WM manipulation periods in our previous study (Kawasaki et al., 2010). We calculated the PSI between a pair of these electrodes at the theta (6 Hz) band during the manipulation periods (for 1 s after the offset of the manipulation cue) compared with the corresponding values recorded during the ITI under the four conditions.

**Figure 2:**
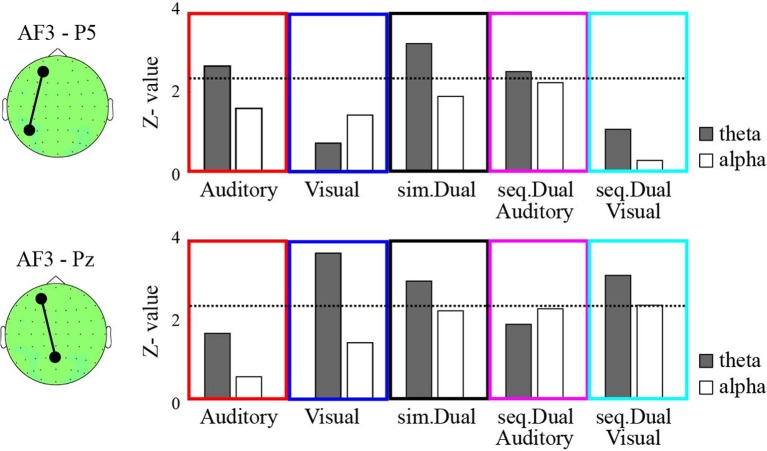
**The *z*-values of the phase synchronization index (PSI) between AF3 and P5 (top) and between AF3 and Pz (bottom) during the manipulation periods compared with the ITI under the auditory-verbal single WM (red), the visual single WM (blue), the simultaneous WM (black), the auditory-verbal manipulation in the sequential WM (magenta), and the auditory-verbal manipulation in the sequential WM (cyan) tasks**. The gray and white bars indicate the *z*-values of the theta and alpha synchronizations, respectively. The dotted lines in each panel denote the threshold value (*P* < 0.01). The topographies on the left show the AF3, P5, and Pz electrodes on the recording montage.

The bootstrapped results revealed that the theta (6 Hz) PSI_AF3−P5_ was significant during the manipulation periods (for 1 s after the offset of the manipulation cue) compared with the corresponding values recorded during the ITI in the auditory-verbal WM, simultaneous WM, and auditory-verbal manipulation of sequential WM (*P* < 0.01; Figure 2). In contrast, the theta PSI_AF3−Pz_ significantly increased in the visual WM, simultaneous WM, and visual manipulation of sequential WM (*P* < 0.01). No significant increases were observed in the theta PSI_AF3−P5_ for the visual WM and the PSI_AF3−Pz_ for the auditory-verbal WM.

Moreover, we analyzed the PSI of the higher-frequency bands (i.e., alpha, beta, and gamma); however, the PSI_AF3−P5_ and the PSI_AF3−Pz_ observed during the auditory-verbal and visual WM conditions were not significantly different between the manipulation periods and the ITI. The PSI results of the alpha (12 Hz) band are shown in Figure 2, as examples.

## Discussion

The present study demonstrated clearly that theta phase synchronization played an important role in selecting and connecting the relevant brain areas during WM manipulation. A previous study showed that the frontal area is significantly synchronized with the parietal areas (i.e., visual regions) or with the temporal areas (i.e., auditory-verbal regions) during the manipulation of visual or auditory-verbal representation, respectively (Kawasaki et al., 2010). In addition, the present study used sequential dual WM tasks, which require manipulation of one modality and maintenance of the other modality, and showed theta phase synchronization only between the relevant brain regions (e.g., fronto-parietal synchronization for the visual manipulation). In contrast, both fronto-parietal and fronto-temporal theta phase synchronizations were observed during the simultaneous dual WM, which requires the manipulation and maintenance of both modalities.

The slow-oscillatory (i.e., theta) synchronization reflects the dynamic, long-range linking of task-relevant brain areas within WM brain networks (Sauseng et al., 2005; Mizuhara and Yamaguchi, 2007; Klimesch et al., 2008; Kawasaki et al., 2010). Previous EEG studies in human have shown that theta phase synchronization between frontal and temporal regions is observed during several WM tasks including encoding, maintenance, and retrieval processes (Sarnthein et al., 1998; Sauseng et al., 2004; Serrien et al., 2004). Moreover, fronto-parietal theta synchronization is enhanced under WM tasks which required high WM load and complex manipulation (Sauseng et al., 2005; Kopp et al., 2006; Payne and Kounios, 2009). The present study also supports the hypothesis that theta synchronization is a requirement of executive function.

Functional theta synchronization is also observed within the resting-state brain network (Von Stein and Sarnthein, 2000; Buzsaki and Draguhn, 2004; Jensen and Colgin, 2007) and long-term memory (especially memory formation) brain networks (Summerfield and Mangels, 2005; Sato and Yamaguchi, 2007). For example, theta synchronization between frontal lobe and hippocampus appeared during long-term memory formation and stimulus encoding (Hasselmo et al., 2002). Moreover, a recent study based on TMS and EEG showed that the resetting of the theta phase propagates directionally from the TMS-targeted areas to the sensory-motor brain areas (Kawasaki et al., 2014). These findings suggest an important role for theta synchronization in large-scale communication and information transfer among distant “task-relevant” brain regions (Fries, 2005; Fell and Axmacher, 2011).

In contrast to global theta synchronization, higher-frequency oscillations would show local synchronizations within circumscribed brain areas during WM (Lutzenberger et al., 2002; Babiloni et al., 2004; Klimesch, 2012), although some studies have reported global beta or gamma phase synchronizations (Tallon-Baudry et al., 2001; Axmacher et al., 2008). Unlike low frequency, the higher-frequency phases are difficult to synchronize across global areas, since such synchronization requires accurate simultaneous activation of areas that are far apart. Indeed, the present study found no significant phase synchronization between brain regions in the high-frequency bands for any WM manipulation. Our previous study, which was performed using the same tasks, showed enhancements of the alpha oscillations in the local brain areas in WM maintenance periods. Moreover, many studies demonstrated the presence of amplitude modulations in the local relevant brain areas during several cognitive tasks (Klimesch et al., 2008), although some studies reported global gamma phase synchronization (Rodriguez et al., 1999; Doesburg et al., 2008). Such high oscillations are assumed to be functionally related to the slow oscillations, such as the coupling between theta phases and gamma amplitudes (Klimesch et al., 2008) and the coupling between theta and beta amplitudes (Kawasaki and Yamaguchi, 2013). The relationships between the global theta and high-frequency phase synchronization in WM should be clarified in future studies.

The finding of modality-specific posterior brain regions and modality-nonspecific frontal regions would be consistent with previous psychological WM models which propose the existence of modality-independent sensory storage buffers (e.g., phonological loop and visuo-spatial sketchpad) and a common central executive (e.g., Scarborough, 1972; Baddeley, 1986; Luck and Vogel, 1997). The overlapped prefrontal cortex are synchronized with not only the visual (parietal) areas but also the auditory-verbal (temporal) areas in the simultaneous dual WM task, which might lead to dual-task interference, that is, degraded performance relative to a single task when two tasks are being performed simultaneously (i.e., psychological refractory period) (Pashler, 1994; Logan and Gordon, 2001). Moreover, the enhancements of frontal activity during the shortened stimulus onset asynchrony between the two tasks in the dual WM tasks would then be the neural factor responsible for the bottleneck in executive functions (Herath et al., 2001; Jiang et al., 2004; Marois and Ivanoff, 2005).

The behavioral data showed that the accuracy rates under the simultaneous dual WM condition were lower than under the other three conditions. This might be consistent with previous findings, which suggest greater difficulty with greater dual-tasking (i.e., psychological refractory period) (Pashler, 1994; Logan and Gordon, 2001). However, it is difficult to compare among conditions, because the accuracy rate was very high, over 90%; that is to say, there is a ceiling effect in our behavioral data. Therefore, there might be a possibility that the results would be different under conditions of increased difficulty.

On the other hand, due to the high accuracy rates, we could accurately identify the theta synchronization by using EEG data from the many trials in which the subjects successfully manipulated the WM representations. However, on this point also, we have no findings on how prominent the synchronization would be at greater task difficulty.

In this study, the theta synchronization might be involved in the manipulation rather than in maintenance of representations. Although the one-modality manipulation periods under the sequential WM condition are defined as not only the manipulation of one modality but also the maintenance of the other, the theta synchronization was observed between only the to-be-manipulated modality-related brain areas (e.g., fronto-parietal theta synchronization under the visual manipulation periods). However, the subjects could have processed the other modality's executive function (e.g., selection of the to-be-manipulated modality and inhibition of the to-be-maintained modality). Moreover, there is a limitation on pure comparison between manipulation and maintenance in our tasks, since manipulation always included maintenance. To address this issue, future study should isolate these functions and compare among them clearly.

### Conflict of interest statement

The authors declare that the research was conducted in the absence of any commercial or financial relationships that could be construed as a potential conflict of interest.
